# “PNP slows down” – linearly-reduced whole body joint velocities and altered gait patterns in polyneuropathy

**DOI:** 10.3389/fnhum.2023.1229440

**Published:** 2023-09-13

**Authors:** Isabelle D. Walz, Sarah Waibel, Vittorio Lippi, Stefan Kammermeier, Albert Gollhofer, Christoph Maurer

**Affiliations:** ^1^Department of Neurology and Neuroscience, Faculty of Medicine, Medical Center, University of Freiburg, Freiburg, Germany; ^2^Department of Sport and Sport Science, University of Freiburg, Freiburg, Germany; ^3^Faculty of Medicine Freiburg, Institute of Digitalization in Medicine, Medical Center, University of Freiburg, Freiburg, Germany; ^4^Department of Neurology, Ludwig Maximilian University, Munich, Germany

**Keywords:** motor control, gait, polyneuropathy, instrumented timed-up-and-go, TUG, joint velocity, whole-body motion capture

## Abstract

**Introduction:**

Gait disturbances are a common consequence of polyneuropathy (PNP) and a major factor in patients’ reduced quality of life. Less is known about the underlying mechanisms of PNP-related altered motor behavior and its distribution across the body. We aimed to capture whole body movements in PNP during a clinically relevant mobility test, i.e., the Timed Up and Go (TUG). We hypothesize that joint velocity profiles across the entire body would enable a deeper understanding of PNP-related movement alterations. This may yield insights into motor control mechanisms responsible for altered gait in PNP.

**Methods:**

20 PNP patients (61 ± 14 years) and a matched healthy control group (CG, 60 ± 15 years) performed TUG at (i) preferred and (ii) fast movement speed, and (iii) while counting backward (dual-task). We recorded TUG duration (s) and extracted gait-related parameters [step time (s), step length (cm), and width (cm)] during the walking sequences of TUG and calculated center of mass (COM) velocity [represents gait speed (cm/s)] and joint velocities (cm/s) (ankles, knees, hips, shoulders, elbows, wrists) with respect to body coordinates during walking; we then derived mean joint velocities and ratios between groups.

**Results:**

Across all TUG conditions, PNP patients moved significantly slower (TUG time, gait speed) with prolonged step time and shorter steps compared to CG. Velocity profiles depend significantly on group designation, TUG condition, and joint. Correlation analysis revealed that joint velocities and gait speed are closely interrelated in individual subjects, with a 0.87 mean velocity ratio between groups.

**Discussion:**

We confirmed a PNP-related slowed gait pattern. Interestingly, joint velocities in the rest of the body measured in body coordinates were in a linear relationship to each other and to COM velocity in space coordinates, despite PNP. Across the whole body, PNP patients reduce, on average, their joint velocities with a factor of 0.87 compared to CG and thus maintain movement patterns in terms of velocity distributions across joints similarly to healthy individuals. This down-scaling of mean absolute joint velocities may be the main source for the altered motor behavior of PNP patients during gait and is due to the poorer quality of their somatosensory information.

**Clinical Trial Registration:**

https://drks.de/search/de, identifier DRKS00016999.

## 1. Introduction

Polyneuropathies (PNP) cover a group of diseases that primarily cause damage to peripheral nerve fibers in symmetric, distal, length-dependent “glove and stocking” distribution (Findling et al., 2018). Affected patients suffer from sensitivity impairments that can manifest in paresthesia, dysesthesia, numbness, and pain in the hands and feet (Hanewinckel et al., 2016; Alam et al., 2017). PNP is also associated with reduced muscular strength (Rotta et al., 2000; Ferreira et al., 2017). This may directly result from damage to motor nerve fibers in specific types of PNP or advanced disease settings or indirectly from a lack of movement due to the aforementioned sensitivity symptoms (Andreassen et al., 2006; Martinelli et al., 2013; Hafsteinsdottir and Olafsson, 2016; Broers et al., 2019).

Functionally speaking, PNP significantly raises the risk of postural instability, meaning balance problems and gait disturbances (Mustapa et al., 2016; Kneis et al., 2020b). For example, in older populations, PNP accounts for around 18% of gait disorders (Pirker and Katzenschlager, 2017) and raises the probability of falling (Cavanagh et al., 1992; Richardson and Hurvitz, 1995; Stolze et al., 2004; Hanewinckel et al., 2017; Findling et al., 2018). PNP can therefore reduce both our everyday mobility and quality of life considerably (Hoffman et al., 2015; Win et al., 2019) and strain healthcare resources (Gordois et al., 2003; Pike et al., 2012; Mengel et al., 2018). Standardized clinical tests usually include gait and balance tasks to assess one’s mobility status and fall risk. Observation of these tests can be refined by motion capture systems enabling specific analysis, e.g., about gait abilities. It is well known that people with PNP habitually walk slower, take shorter steps, and reveal more gait variability than age-matched healthy individuals (Wuehr et al., 2014; Hanewinckel et al., 2017; Marshall et al., 2017; Kneis et al., 2020a). However, it remains unclear whether the altered gait, especially in terms of slowness, is caused by the primary deficit, i.e., damaged peripheral nerve structures, or whether it is a secondary strategy to prevent accidents such as falls (Courtemanche et al., 1996; Dingwell et al., 2000; Dingwell and Cavanagh, 2001; Menz et al., 2004; Wuehr et al., 2014). As PNP makes evident, damage to peripheral nervous structures leads to proprioceptive impairments (Hanewinckel et al., 2016; Findling et al., 2018). Reliable proprioceptive feedback is essential for precise posture control, as it contains information about alterations via tendon or muscle lengths and joint angles (Allum et al., 1998; Dietz, 2002; Masani et al., 2003; Shaffer and Harrison, 2007). Disturbances in the proprioceptive signals thus imply inaccurate motor control mechanisms (Dietz et al., 2002; Pearson, 2004). In PNP, this somatosensory deficit is believed to promote the aforementioned balance and gait disturbances. However, its exact interrelation with the slowing of gait remains unclear (Courtemanche et al., 1996; Dingwell et al., 2000; Menz et al., 2004; Wuehr et al., 2014). Besides gait and balance, the functional performance of upper limbs also suffers from PNP. There is evidence of less accuracy and slower execution speed during goal-directed arm movements (Hondzinski et al., 2010), as well as reduced functional hand performance during fine motor tasks compared to healthy individuals, while strength capacity seems unaffected (Kender et al., 2022). Until now, there has been no known whole-body gait movement analysis in PNP considering both upper and lower-body motor behavior. We assume that altered motor pattern in the lower limbs while walking is also reflected in the upper limbs (Wannier et al., 2001; Meyns et al., 2013; Pearcey and Zehr, 2019).

Our approach in the present study is to capture the whole-body movement of PNP patients in a standardized mobility test acknowledged as clinically relevant, i.e., the Timed Up and Go (TUG) test (Salarian et al., 2010; Christopher et al., 2019). The TUG test covers essential demands of daily living (standing up and sitting down, accelerating and decelerating walking, turning around) and, thus, stimulates different body systems responsible for posture stability, coordinated movements, and force development. TUG was originally done at a preferred movement speed (Podsiadlo and Richardson, 1991), operating as a reliable and approved sign of vitality (Studenski et al., 2011; Perera et al., 2016). For a more differentiated perspective of PNP-related sensorimotor impairments, we will add two further TUG conditions that challenge patients’ executive resources: executing TUG while counting backward to provoke cognitive-motor interference (Montero-Odasso et al., 2012; Montero-Odasso and Hachinski, 2014; Bayot et al., 2018; Schniepp et al., 2019), and at a fast movement speed to assess acceleration capacities associated with disability and functional reserve capacity (Wuehr et al., 2014; Artaud et al., 2015; Middleton et al., 2015). For motion behavior analysis, we will extract common gait parameters and additionally focus on joint velocities across the whole body in comparison to matched healthy control individuals. As the first step, we aim to verify that the TUG performance of PNP patients falls below that of healthy individuals. We assume that PNP-related gait alterations will reflect the poorer performance level in each TUG condition. Furthermore, we hypothesize that velocity across the whole body can characterize PNP-specific motion patterns depending on the TUG condition and joints analyzed. We believe that our approach will enable us to derive additional velocity-based parameters that will help us better understand motor behavior and movement organization in neurological diseases.

## 2. Materials and methods

### 2.1. Participants

We enrolled 20 patients with clinically confirmed polyneuropathy symptoms (PNP) and 20 healthy control participants (control group, CG) matched to patients’ age, sex, height, and weight. Exclusion criteria for the PNP group cover comorbidities that interact with gait and balance abilities. For CG, we excluded any disease that could be related to PNP symptoms or interfere with gait or balance performance. Patients’ PNP symptoms were objectified by testing reflexes, vibration sense, joint position sense, temperature, and pain sensation in the lower extremities (Liniger et al., 1990; Pestronk et al., 2004; Kneis et al., 2020b). All participants underwent detailed anamnesis, including chronic or acute diseases requiring treatment. Furthermore, we asked for the maximum walking distance as well as the number of falls during the last year and estimated the fear of falling via the validated Falls Efficacy Scale – International (FES-I) (Delbaere et al., 2010). We also clinically assessed mobility performance by applying a common test, i.e., the Performance Oriented Mobility Assessment (Tinetti POMA), with nine items for balance (score 0–16) and eight items for gait (score 0–12): a lower score indicates a higher risk of falling (Tinetti, 1986).

This study was approved by the Ethics Committee of the University of Freiburg (no. 68/19) and conducted according to the Declaration of Helsinki (German Register of Clinical Trials no.: DRKS00016999). Written informed consent was obtained from all individual participants included in the study.

### 2.2. Assessments

All participants performed the Timed Up and Go (TUG) test in three different conditions twice: performing TUG first at preferred movement speed (preferred condition), secondly while executing an additional cognitive task [counting backward in steps of two (Al-Yahya et al., 2011); dual-task condition], and thirdly as fast as possible without running, meaning one or both feet always in ground contact (fast condition, test instruction: “walk as fast as safely possible”). The instructions were standardized. Participants walked wearing their own footwear. Each TUG condition was performed twice.

### 2.3. Motion capture

All movements during TUG execution were recorded via a markerless vision-based motion capture system, i.e., The Captury (The Captury GmbH, Saarbrücken, Germany). It uses a visual hull and background subtraction method to estimate the subject’s silhouette. Body movements are tracked by 12 cameras at a 100 Hz sampling rate and resolution of ~1 mm. An automatic scaling process fits a skeleton into the subject (up to 60 s). The system calculates precise position data of the whole body, represented by specific joints, e.g., wrist, elbow, shoulder, hip, knee, and ankle, and center of mass (COM) estimation (Kuhner et al., 2017; Harsted et al., 2019).

### 2.4. Data processing

A custom build MATLAB^™^ (R2019b; MathWorks, Natick, Ma) program was used for data processing. For analysis, we relied on the mean values of the two trials per TUG condition. We identified the duration (s) needed to complete each TUG trial (TUG time). Furthermore, we extracted three sequences from the TUG: walk 1 [(s), walk between the stand-up and turning task], turning (s), and walk 2 [(s), walk between turning- and turn-to-sit-task]. For gait-specific analysis, steps during the walking sequences (walk1 + 2) were detected using the ankle speed: steps begin and end if the ankles’ speed approaches zero (threshold = 5.5 cm/s). Turning and turn-to-sit sequences were identified by shoulder axis rotation (>20°). The thresholds were determined based on own datasets for validation. Step detection serves to calculate these gait parameters: step time (s) and step length (cm), as well as step width (cm).

Velocity measures were calculated using the mean values of walk 1 and walk 2. COM velocity refers to space coordinates and thus represents gait speed (cm/s). As the velocity between left and right joints was not asymmetric, individual joint velocities (cm/s) were presented as mean (wrist, elbow, shoulder, hip, knee, and ankle) and calculated with respect to COM during walking. We then derived mean joint velocities (per joint and across all joints) for each group and velocity ratios between groups.

### 2.5. Statistical analysis

For statistical analysis, IBM SPSS Statistics for Windows, version 26.0 (IBM Corp., Armonk, NY, United States), and for data visualization, RStudio, version 4.0.3 (RStudio, PBC, Boston, United States) was used. Descriptive statistics are reported as median with a 25–75 percentile range. Participants’ characteristics were analyzed using T-Test, Pearson-Chi-Quadrat, and Man-Whitney-U tests. Shapiro–Wilk test was used to test for normal distribution of TUG times, gait parameters, and COM velocity (all parameters were normally distributed, except turning and walking 2 times in PNP). TUG times, gait parameters, and COM velocity were analyzed separately using repeated measures ANOVA, with the condition as the dependent variable (Schmider et al., 2010). Bonferroni was used as a post-hoc test.

Joint velocities were log-transformed to achieve normal distribution, as the original data was skewed to the right. To assess joint velocities, we applied the repeated measures multivariate analysis of variance (MANOVA) to evaluate the complexity of variable relationships by considering two dependent variables (joints and TUG condition) and group designation as independent (repeated measures) variables. As a post-hoc test for TUG condition, we used the Bonferroni, for joints, the Tukey’s Honest Significant Difference (HSD) test. To demonstrate the relations between joint velocities and gait speed (COM), we conducted a correlation analysis. We chose the ankle (as the most distal joint) and hip (as the most proximal joint) to visualize joint behavior relative to gait speed. Spearman-Rho was used to correlate velocity ratios (wrist-, elbow-, shoulder-, hip-, knee-ankle ratio, and overall velocity ratio) with maximum walking distance, fear of falling (FES-I), and mobility performance (Tinetti POMA).

## 3. Results

No adverse events occurred during the tests, and all participants performed all test conditions. We included data from *N* = 40 participants (20 PNP:20 CG) in our analysis. The comparative groups PNP and matched CG exhibited similar anthropometric parameters (Table 1). Participants’ characteristics revealed significant group differences in fall incidence, maximum walking distance, fear of falling (FES-I), mobility performance (Tinetti POMA; Table 1), medicine intake, and chronic disabilities (Supplementary Table S1). All included patients had relevant PNP symptoms (Table 2).

**Table 1:** Participants’ characteristics.

	PNP	matched CG	*p*-value
	*n* = 20	*n* = 20	
**Age** mean ± SD	60.7 ± 13.9	60.4 ± 14.7	0.939^1^
**Sex** (m:f) *N* (%)	15:5 (75:25)	15:5 (75:25)	1.000^2^
**BMI** (kg/m^2^) mean ± SD	26.9 ± 5.2	25.2 ± 3.9	0.264^1^
**Falls (past year)** *N*	32	2	**0.001** ^3^
Faller / non-faller *N* (%)	13(65)/7(35)	2 (10)/18 (90)	
**FES-I (16–64 Points)** mean (range)^a^	22.1 (16–38)	17.4 (16–20)	**0.006** ^3^
Low concern (16–19) *N* (%)	9 (45)	16 (80)	
Moderate concern (20–27) *N* (%)	6 (30)	4 (20)	
High concern (28–64) *N* (%)	5 (25)	0 (0)	
**Tinetti POMA (0–28 Points)** mean (range)^b^	22.6 (10–28)	27.8 (27–28)	**<0.001** ^3^
Moderate risk of falling (19–24) *N* (%)	6 (30)	0 (0)	
High risk of falling (10–19) *N* (%)	5 (25)	0 (0)	
**Maximum walking distance (km)** mean (range)	6.8 (0.5–18)	12.8 (2–50)	**0.008** ^3^

PNP, polyneuropathy patients; matched CG, matched healthy control group; SD, standard deviation; ^1^ *T*-Test; ^2^ Pearson-Chi-Quadrat; ^3^ Man-Whitney-U.

^a^ Classification from Delbaere et al. (2010); ^b^ Classification from Tinetti et al. (1986); significant differences (*p* < 0.05) between groups are marked in bold.

**Table 2:** PNP-specific characteristics.

	PNP
	*n* = 20
**PNP entity** *N* (%)	
CIPN	4 (20)
CIDP	15 (75)
Not classified	1 (5)
**PNP specific treatment** *N* (%)	
Rituximab	2 (10)
IVIg	14 (70)
**PNP symptoms** *N* (%)	
Reduced vibration sense MJ/ML^#^	15 (75)/15 (75)
Reduced joint position sense^+^	9 (45)
Reduced temperature sensation*	12 (60)
Reduced pain sensation*	7 (35)
Loss of reflexes AT/PT	10 (50)/6 (30)
Reduced reflexes AT/PT	4 (20)/8 (40)

PNP, polyneuropathy patients; CIPN, chemotherapy induced polyneuropathy; CIDP, chronic inflammatory demyelinating polyneuropathy; IVIg, intravenous immunoglobulin infusions; ^#^vibration sense was measured on the Metatarsophalangeal joint (MJ) and Malleolus lateralis (ML) by the Rydell-Seiffer tuning fork [scale ranging from 0/8 (no sensitivity) to 8/8 (highest sensitivity)], value ≤ 4 was rated as reduced; ^+^measured on second toe, ≥ 3 failures out of 10 trials in random order; *measured on arch, ≥ 3 failures out of 10 trials in random order. AT, Achilles tendon; PT, Patella tendon.

### 3.1. TUG times and TUG-related gait parameters

PNP patients performed slower across all TUG conditions than their matched CG: The PNP group needed more time to complete TUG (4.2 s at preferred, 2.8 s at dual-task, and 1.7 s at fast condition), walked slower (29 cm/s slower gait speed at preferred, 14.8 cm/s at dual-task, and 14.1 cm/s at fast condition) with prolonged step time (5% at preferred, 6% at dual-task, and 6% at fast condition). Patients also took shorter steps during preferred (5.2 cm) and dual-task (5.1 cm) walking, while in the fast condition, the step lengths differed marginally (2.1 cm) (Table 3).

**Table 3:** Descriptive illustration of the TUG parameters for the different TUG conditions of polyneuropathy patients (PNP) and matched control group (CG).

Parameter	TUG condition	PNP median (IQR)	matched CG median (IQR)
		*n* = 20	*n* = 20
TUG time (s)	P	13.4 (10.2–15.0)	9.2 (8.4–10.7)
	DT	13.0 (10.8–16.5)	10.2 (8.8–11.2)
	F	8.2 (6.2–10.9)	6.5 (5.8–7.0)
Walk 1 (s)	P	4.4 (3.3–4.6)	2.8 (2.6–3.5)
	DT	4.4 (3.4–4.7)	3.3 (2.8–3.7)
	F	2.4 (1.9–3.4)	1.9 (1.6–2.2)
Turning (s)	P	1.7 (1.4–2.0)	1.3 (1.2–1.4)
	DT	1.7 (1.4–2.3)	1.3 (1.2–1.4)
	F	1.1 (0.9–1.7)	0.9 (0.9–1.0)
Walk 2 (s)	P	3.6 (3.0–4.4)	2.8 (2.4–3.2)
	DT	3.8 (3.3–4.9)	3.1 (2.7–3.4)
	F	2.3 (1.8–3.1)	1.9 (1.6–2.0)
Gait speed (COM) (cm/s)	P	80.7 (73.7–100.5)	109.7 (100.4–119.1)
	DT	81.9 (66.1–98.7)	96.7 (89.4–115.2)
	F	134.7 (96.5–166.2)	148.8 (134.1–175.0)
Step time (s)	P	0.60 (0.57–0.65)	0.57 (0.54–0.60)
	DT	0.65 (0.60–0.74)	0.61 (0.58–0.66)
	F	0.50 (0.48–0.55)	0.47 (0.42–0.50)
Step length (cm)	P	53.8 (41.2–60.4)	59.0 (56.2–64.1)
	DT	51.3 (43.1–59.9)	56.4 (53.3–62.6)
	F	61.2 (50.1–70.6)	63.3 (59.5–68.3)
Step width (cm)	P	14.5 (10.9–16.8)	13.7 (11.7–16.0)
	DT	13.1 (11.2–17.8)	13.3 (11.3–16.1)
	F	14.1 (13.0–17.8)	14.4 (12.8–16.3)

IQR, interquartile range (25–75 percentile); TUG, timed up and go test; PNP, polyneuropathy patients; matched CG, matched healthy control group; COM, center of mass; P, preferred condition, DT, dual-task condition; F, fast condition.

We detected significant group effects for all TUG times (overall, walk 1, walk 2, turning) and the TUG-related gait parameters gait speed, step time, and length (*p* < 0.001 respectively), except for step width (*p* = 0.568). The TUG condition showed a significant effect on all TUG parameters (excluding step width). Post-hoc tests revealed a significant difference between fast and preferred and between fast and dual-task conditions for all TUG parameters (*p* < 0.001). The step time, additionally, differed between preferred and dual-task (*p* < 0.001). We found no interaction between group designation and TUG condition (Table 4).

**Table 4:** Repeated measures ANOVA results for TUG times and TUG-related gait parametersPL.

Parameter	Group (PNP, matched CG)	TUG condition (P, DT, F)	Group*TUG condition
TUG time (s)	F = 72.84 ***p* < 0.001**	F = 22.77 ***p* < 0.001**	F = 0.75 *p* = 0.476
walk 1 (s)	F = 46.71 ***p* < 0.001**	F = 28.64 ***p* < 0.001**	F = 0.69 *p* = 0.506
turning (s)	F = 40.69 ***p* < 0.001**	F = 9.51 ***p* < 0.001**	F = 0.46 *p* = 0.637
walk 2 (s)	F = 60.04 ***p* < 0.001**	F = 21.75 ***p* < 0.001**	F = 0.38 *p* = 0.685
Gait speed (cm/s)*	F = 51.35 ***p* < 0.001**	F = 40.29 ***p* < 0.001**	F = 0.67 *p* = 0.516
Step time (s)	F = 32.70 ***p* < 0.001**	F = 122.86 ***p* < 0.001**	F = 0.06 *p* = 0.942
Step length (cm)	F = 60.04 ***p* < 0.001**	F = 7.21 ***p* = 0.001**	F = 0.97 *p* = 0.383
Step width (cm)	F = 0.33 *p* = 0.568	F = 0.96 *p* = 0.385	F = 0.54 *p* = 0.586

PNP polyneuropathy patients; matched CG matched healthy control group; TUG timed up and go test; COM center of mass; P preferred condition DT dual-task condition; F fast condition; *gait speed = COM velocity. Significant *p*-values <0.05 are marked in bold.

### 3.2. Joint velocities

**Figure 1:**
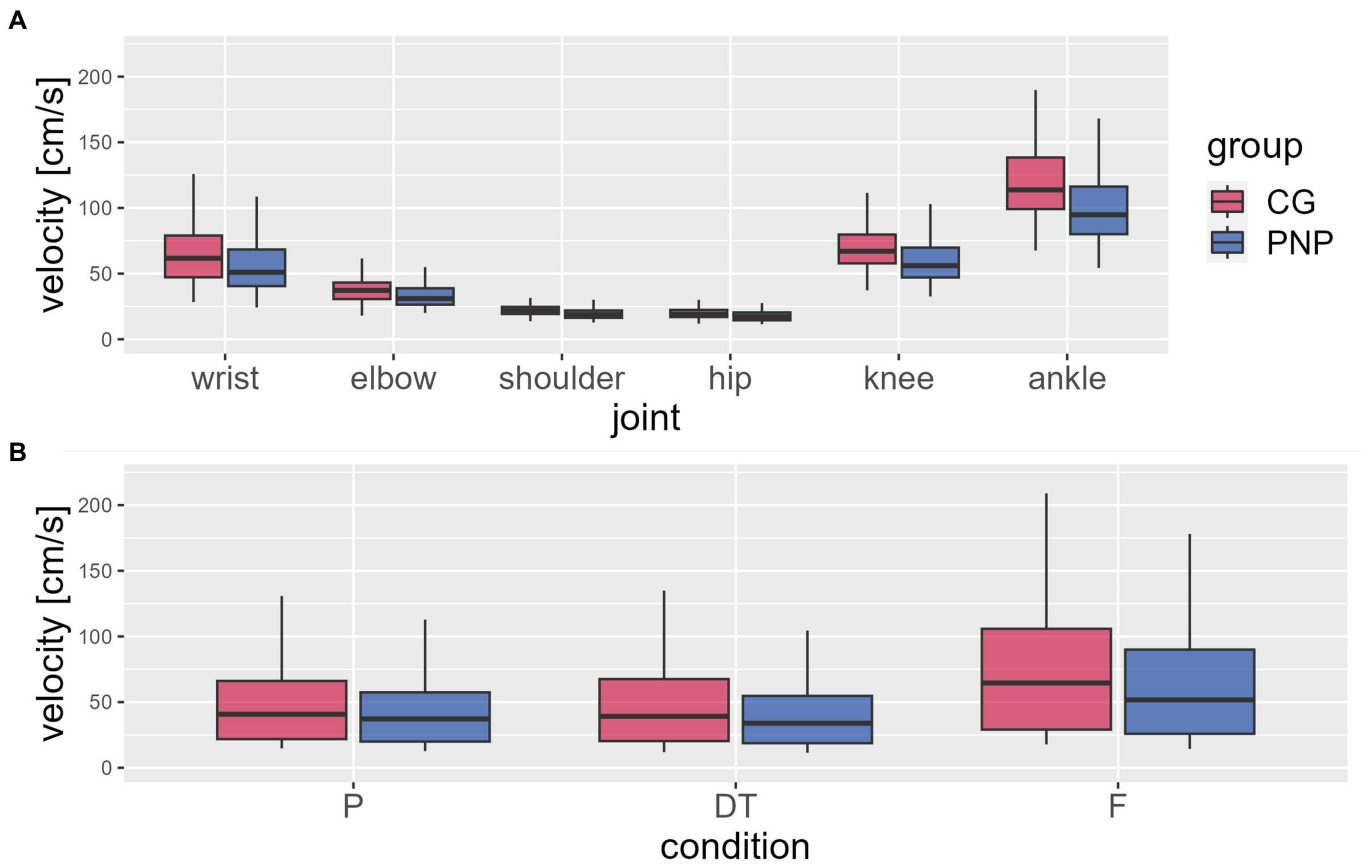
Mean joint velocities. The figure shows the mean joint velocities relative to the center of mass (in body coordinates) (y-axis) extracted from the waking sequences during the timed up and go test (TUG) per group [CG (red), control group; PNP (blue), patients with peripheral neuropathy]: **(A)** average value per joint (x-axis) across all conditions and **(B)** per condition (P, preferred; DT, dual-task; F, fast condition) across joints. Boxplots showing the lower quartile (25th percentile), median (50th percentile), upper quartile (75th percentile), and degree of dispersion as 95% confidence interval (95% CI) (whiskers).

Analysis of joint velocities (MANOVA) revealed a significant influence of the factors group designation (PNP, CG; *F* = 123.9, *p* < 0.001, η^2^ = 0.266, Figures 1A, B), joint (wrist, elbow, shoulder, hip, knee, ankle; *F* = 1152.6, *p* < 0.001, η^2^ = 0.944, Figure 1A), and TUG condition (preferred, dual-task, fast; *F* = 218.1, *p* < 0.001, η^2^ = 0.561, Figure 1B).

The PNP group revealed a significantly slower velocity than CG (−7.9 cm/s, 95%CI −9.1 to −6.6) across all conditions and joints. No group interaction was found for either joint or TUG condition (Figure 1B).

Velocities varied depending on joints (across groups and conditions), with higher velocities at distal joints compared to proximal joints (Figure 1A). For example, the ankle, the most distal joint in this analysis, displayed 80% higher velocity than the hip, the most proximal joint (Figure 1A). Post-hoc-test of joint velocities revealed significant differences in all pairwise joint comparisons (*p* ≤ 0.001) except for the comparison of knee and wrist (*p* = 0.082) (See Table 5).

**Table 5:** Joint velocities of PNP and matched CG in body coordinates relative to COM.

Condition		Joint	PNP median (IQR)	matched CG median (IQR)
			*n* = 20	*n* = 20
Preferred	Velocity (cm/s)	Wrist	47.8 (40.5–53.4)	56.9 (46.9–63.8)
		Elbow	30.8 (26.4–34.1)	34.9 (32.0–38.5)
		Shoulder	18.6 (16.0–20.7)	20.8 (18.7–22.3)
		Hip	16.6 (14.1–18.8)	17.9 (16.2–20.6)
		Knee	53.5 (45.2–61.7)	61.9 (59.7–72.5)
		Ankle	85.3 (77.2–103.7)	108.2 (99.1–115.9)
Dual-task	Velocity (cm/s)	Wrist	48.3 (36.6–53.7)	55.1 (43.6–60.7)
		Elbow	28.7 (23.5–31.7)	33.6 (29.4–37.3)
		Shoulder	18.2 (15.4–19.0)	20.4 (18.0–21.8)
		Hip	15.6 (13.2–18.5)	16.7 (15.6–19.0)
		Knee	51.3 (42.9–55.7)	56.0 (49.7–67.9)
		Ankle	85.1 (71.5–100.0)	98.7 (89.8–114.4)
Fast	Velocity (cm/s)	Wrist	79.8 (60.4–93.0)	88.3 (70.1–112.9)
		Elbow	43.2 (33.7–50.9)	49.8 (40.5–63.9)
		Shoulder	22.3 (19.0–27.2)	25.7 (23.3–30.0)
		Hip	20.9 (17.6–24.6)	22.9 (19.9–25.9)
		Knee	78.9 (62.7–97.8)	82.0 (776.7–104.7)
		Ankle	136.6 (96.5–154.7)	148.6 (133.9–163.0)

PNP, polyneuropathy patients; matched CG, matched healthy control group; IQR, interquartile range (25–75 percentile).

Velocities varied depending on the TUG condition (across groups and joints): the fast condition revealed higher velocities than the preferred (20.7 cm/s, 95%CI 17.9–23.5, *p* < 0.001) and dual-task condition (23.4 cm/s, 95%CI 20.6–26.2, *p* < 0.001), moreover, the preferred condition revealed higher velocities than the dual-task condition (*p* = 0.012) (Figure 1B).

### 3.3. Correlation analysis

**Figure 2:**
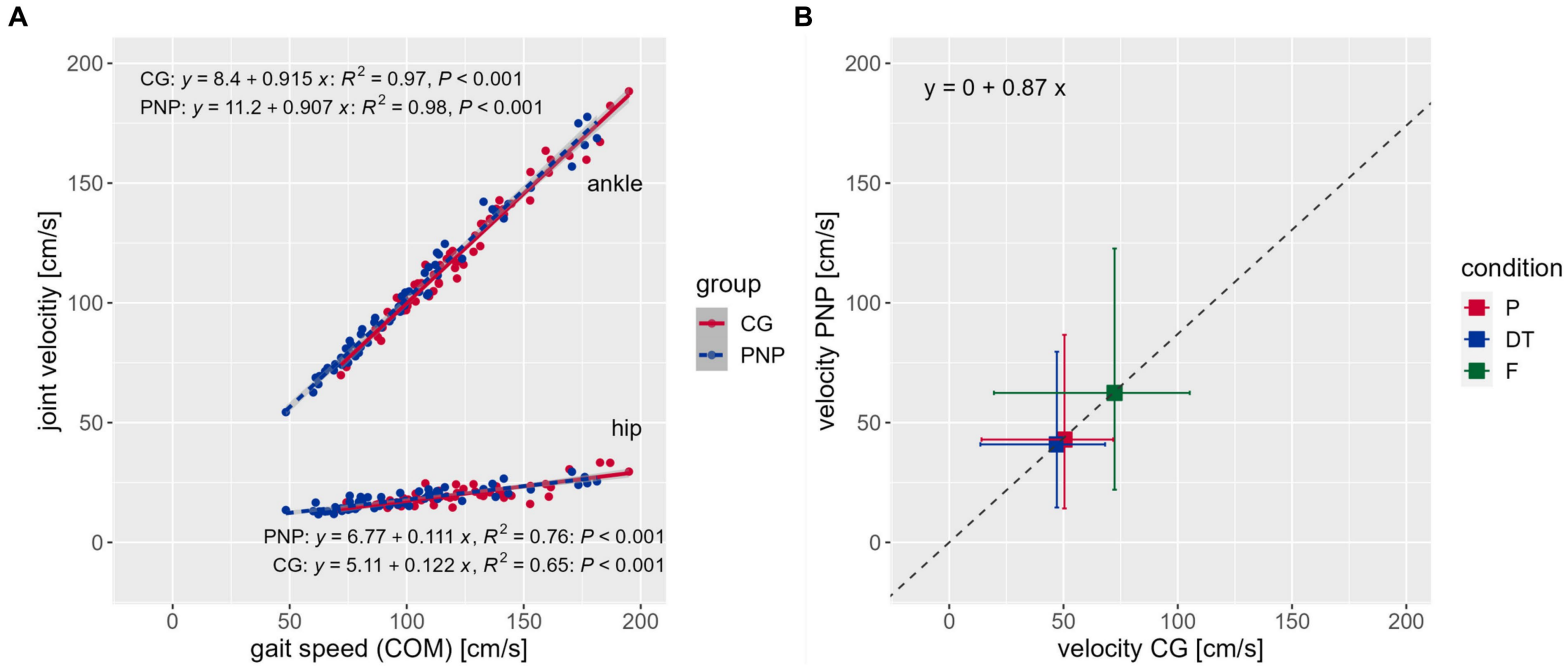
Correlations. **(A)** Correlations between the center of mass (COM) representing gait speed (x-axis) and joint velocity (y-axis) across conditions exemplarily for the ankle (top) and hip (bottom) joint velocities per group [CG (red), control group; PNP (blue), patients with peripheral neuropathy], including the regression equation, R-squared and value of p, respectively. **(B)** Displays the mean velocity ratio of 0.87 between groups (see regression equation). Boxes and whiskers show the mean velocities (box) and standard deviation [horizontal whiskers for CG, vertical whiskers for PNP) of the CG (x-axis) against the PNP group (y-axis) per condition (P (red), preferred; DT (blue), dual-task; F (green), fast condition] across joints.

Correlation analysis between gait speed (COM velocity) and all joint velocities within groups revealed that joint velocities and gait speed are positively interrelated in individual subjects (Figure 2A, exemplarily for ankle and hip velocities, see Supplementary Figure S1 for all joint velocities) with a 0.87 mean velocity ratio between groups (Figure 2B). This means that PNP joint velocities are 13% slower than the CG’s regardless of joint and condition.

Our PNP correlation analysis showed that the overall velocity ratio correlated negatively with the fear of falling (FES-I, *r*^2^ = −0.636, *p* = 0.003) and positively with mobility performance (Tinetti POMA, *r*^2^ = 0.583, *p* = 0.007). Hip, knee, and ankle ratios correlated positively with self-reported maximum walking distance (hip, *r*^2^ = 0.562, *p* = 0.010; knee, *r*^2^ = 0.575, *p* = 0.008; ankle, *r*^2^ = 0.556, *p* = 0.011), while the overall velocity ratio did not correlate significantly (velocity ratio, *r*^2^ = 0.401, *p* = 0.080). This indicates that a stronger fear of falling and poorer mobility were associated with a slower gait speed in the patient group (Supplementary Figure S2).

## 4. Discussion

The main objective of the present study was to analyze the motion patterns of PNP patients along the body axis for a deeper understanding of altered motion behavior caused by PNP. We assessed PNP patients’ performance in a clinically relevant functional test, i.e., the Timed-Up-and-Go test (TUG), by comparing gait-related parameters and joint-velocity profiles across the whole body to a group of matched healthy individuals. As hypothesized, PNP patients performed TUG slower than the control group during all test conditions, i.e., at preferred and fast movement speed and while executing a cognitive task (dual-task condition). More specifically, we found a PNP-related slowed gait pattern during TUG execution determined by reduced gait speed, shorter steps, and prolonged step time, as well as lower mean velocities in all other measured body joints. Interestingly, the slowing factor of PNP patients’ joint velocities was independent of the respective joint and, therefore, individual mean joint velocity, which tends to vary across the body. Moreover, this factor applied to each TUG condition similarly and amounted, on average, to 0.87 compared to the healthy control group.

As expected, PNP patients’ overall TUG performance fell below that of the healthy control group across all conditions. Patients needed approximately 20% more time to complete TUG independently of the test condition. By extracting gait-related parameters, we detected a slowed walking pattern in PNP (preferred: 80.7 cm/s; fast: 134.7 cm/s) compared to the CG (preferred: 109.7 cm/s; fast: 148.8 cm/s) determined by shorter steps (preferred: 54 cm vs. 59 cm) and prolonged step time (preferred: 0.60 s vs. 0.57 s). These results concur with other studies investigating PNP-related gait impairments (Fernando et al., 2013; Wuehr et al., 2014; Marshall et al., 2017), identifying 139 cm/s for fast walking (Wuehr et al., 2014), and 93–110 cm/s for preferred walking in PNP patients (Menz et al., 2004; Wuehr et al., 2014; Marshall et al., 2017). In contrast to other studies (Petrofsky et al., 2005; Brach et al., 2008; Wuehr et al., 2014; Brown et al., 2015), we failed to observe a wider gait base in PNP patients than in healthy individuals. Basically, the TUG condition significantly affected TUG performance in both groups in a similar way but at a different speed level.

These findings also apply to joint velocity profiles in PNP compared to CG. As with gait-related parameters, we used the walking sequences of the TUG to extract the mean joint velocities of ankles, knees, hips, shoulders, elbows, and wrists in body coordinates, respectively. Across the whole body, PNP patients reduced, on average, their joint velocities with a factor of 0.87 compared to CG. Interestingly, the velocity reductions occurred independently of the respective joint or test condition. This means that in each of the three test conditions, the average joint velocity of each joint was reduced by 13% in PNP patients compared to healthy individuals. Furthermore, joint velocities (of PNP and CG) were in a linear relationship to each other, and gait speed (COM velocity in space) referred to the respective test condition (Figure 2B). We deduce that despite PNP-related slowing, patients maintained movement patterns in terms of velocity distributions across joints similarly to healthy individuals.

PNP affects the peripheral nerves in a distal symmetric distribution that results in impaired somatosensory information. Especially proprioceptive feedback is essential for posture stability (Dietz, 2002) as proprioceptors provide continuous feedback about joint positions and velocity, thus facilitating our orientation in space with respect to the ground and the different body segments (Lackner and DiZio, 2005; Shaffer and Harrison, 2007). The proprioceptors’ signal carries velocity information relying on changes in lengths (tendons or muscles) or the joint angles that are primarily used to control motion accurately (Allum et al., 1998; Dietz, 2002; Masani et al., 2003; Shaffer and Harrison, 2007). Because of their proprioceptive deficit, PNP patients often suffer from postural instability, which manifests in balance problems (Horlings et al., 2008; Sawacha et al., 2009; Brown et al., 2015; Kneis et al., 2016, 2020b; Mustapa et al., 2016) and gait disturbances (Allet et al., 2008; Sawacha et al., 2009; Shin et al., 2021) and may raise the risk of falls (Stolze et al., 2004; Allet et al., 2008). Patients often experience a loss of muscular strength also due to the PNP-induced impaired sensorimotor interplay (Andreassen et al., 2006; Martinelli et al., 2013; Ferreira et al., 2017). A slower gait speed, including shorter steps and longer step times, may thus become manifest from a diminished propulsion capacity, but it may also follow PNP’s safety management (Dingwell et al., 2000; Mustapa et al., 2016; Findling et al., 2018). Slower, shorter steps imply a relatively higher proportion of the double support phase associated with greater stability (Williams and Martin, 2019). Vice versa, minimizing the support surface during the single-leg stance phase requires more effort to maintain balance, thus challenging the postural system (Williams and Martin, 2019). Furthermore, reducing movement speed may give patients more time to generate an adequate response (Williams and Martin, 2019) and thus contribute to movement accuracy despite the proprioceptive deficit. We are assuming that PNP patients’ slower gait implies the slowdown of upper limbs’ joint velocities according to the hypothesis of strong linear velocity interlimb coupling (Orsal et al., 1990). As mentioned above, our PNP patients showed velocity distributions across joints resembling those of healthy individuals, which corresponds to the manifestation of PNP-induced nerve damage -that is, the symmetrical pattern across extremities.

Despite PNP patients’ reduced overall velocity, we conclude that velocity control mechanisms are largely intact in PNP (Zehr and Duysens, 2004; Meyns et al., 2013). We even propose that slowing movement velocity is potentially a secondary compensatory safety strategy rather than one triggered by the primary physiological deficit. We base this assumption on the fact that our PNP patients were able to adjust their movement speed situationally like healthy individuals but move within a lower individual speed zone. For example, patients increased their movement speed when asked to, as in the fast TUG condition. Furthermore, the concurrent execution of a cognitive task led to the slowing down of movements in PNP to the same extent as in healthy individuals, but again at a lower level. There is evidence that multi-task conditions require an allocation of attentional resources to each task that often results in slowed movement speed (Hausdorff et al., 2008; Montero-Odasso et al., 2012). In general, PNP patients may require greater effort in order to allocate more cognitive resources than healthy individuals to generate well-coordinated movements despite their PNP-related impairments (Courtemanche et al., 1996). We, therefore, suggest that PNP patients scale their postural control strategy along with the quality of the sensory signals they receive. This supposition is supported by findings of studies addressing stance control in PNP patients. We found that the postural behavior of PNP patients is modifiable by an exercise intervention; that people with PNP performed a sensory reweighting and an adjustment of velocity control towards the postural behavior of healthy individuals (Kneis et al., 2019, 2020b).

Our assumption of PNP patients’ preventive safety strategy is highlighted by the correlation between their individual slowing factor with their fear of falling (FES-I), mobility performance (Tinetti POMA), and self-reported maximum walking distance. We suggest that the individual slowing factor measures the degree of PNP mobility impairment.

## 5. Limitations

The 3-meter walking sequences of the TUG test cover a relatively short distance for gait analysis, encompassing both acceleration and deceleration phases. While we obtained plausible values for the extracted gait parameters, our experimental setup did not adhere to common guidelines for gait analysis. Specifically, it deviated from the standard practice of continuous walking over a minimum distance of 10 to 20 strides, as outlined in the literature (Hollman et al., 2010, 2011). Furthermore, our step detection methodology relied on velocity thresholds derived from our own validation work, rather than direct force plate measurements, which are considered the gold standard (Bilney et al., 2003; Steinert et al., 2019). These factors, coupled with the limited sample size, emphasize the exploratory nature of our research, and underscore the need for a cautious interpretation of the results. The diagnostic utility of the TUG test lies in its multidimensional assessment of functional performance. However, in this study, we deliberately focused only on the walking sequence of the TUG test, excluding other motion tasks such as standing up, turning, and sitting down. These additional sequences are vital for a comprehensive understanding of the significance of the TUG test. In the future, we plan to incorporate these sequences into our motion analysis. Moreover, we will also extract additional biomechanical parameters such as joint torques and moments, offering a more comprehensive view of motor behavior during the TUG test.

## 6. Conclusion

Our results indicate that PNP patients reduce all their mean joint velocities similarly, resulting in a patient-specific slowness factor (on average 13%) compared to healthy individuals, regardless of joint or condition. This slowing of all joints in body coordinates correlates strongly with the individual gait speed in space coordinates (COM velocity). We assume that this global slowing is caused by the reduced quality of proprioceptive signals. We maintain that PNP patients’ altered gait, as assessed by mean absolute joint velocities, is not only determined by the obvious balance requirements leading to smaller and slower steps but that it results from a shift in general speed of an otherwise undisturbed whole body motion pattern.

## Data availability statement

The raw data supporting the conclusions of this article will be made available by the authors, without undue reservation.

## Ethics statement

The studies involving humans were approved by Ethics Committee of the University of Freiburg, Germany. The studies were conducted in accordance with the local legislation and institutional requirements. The participants provided their written informed consent to participate in this study.

## Author contributions

IW, SW, and CM drafted the manuscript and analyzed and interpreted the data. IW and CM designed the study. IW recruited patients and collected and analyzed data. VL wrote the code for MATLAB and analyzing the TUG parameters. All authors contributed to the article and approved the submitted version.

## Funding

IW, SW, and CM were partially funded by the European Union’s Horizon 2020 research and innovation program (grant agreement no. 769574) and the European Union’s Horizon 2021 research and innovation program (grant agreement no. 101057747). We acknowledge the funding of the article processing charge by the Open Access Publication Fund of the University of Freiburg.

## Conflict of interest

The authors declare that the research was conducted in the absence of any commercial or financial relationships that could be construed as a potential conflict of interest.

## Publisher’s note

All claims expressed in this article are solely those of the authors and do not necessarily represent those of their affiliated organizations, or those of the publisher, the editors and the reviewers. Any product that may be evaluated in this article, or claim that may be made by its manufacturer, is not guaranteed or endorsed by the publisher.
