# Supplementary material for: “PNP slows down” – linearly-reduced whole body joint velocities and altered gait patterns in polyneuropathy
Source: Front Hum Neurosci. 2023 Sep 13;17:1229440. doi: 10.3389/fnhum.2023.1229440 (PMC10534044; doi:10.3389/fnhum.2023.1229440)
Supplement: Supplementary file 1 [file Data_Sheet_1.docx]

Supplementary Material

“PNP slows down” – linearly-reduced in whole body joint velocities and altered gait patterns in polyneuropathy

Isabelle D. Walz, Sarah Waibel, Vittorio Lippi, Stefan Kammermeier, Albert Gollhofer and Christoph Maurer*

*** Correspondence:** Prof. Dr. med Christoph Maurer: christoph.maurer@uniklinik-freiburg.de

# Supplementary Figures and Tables

Supplement Table 1. Medicine intake and chronic disabilities in PNP patients and healthy matched CG.

|  | **PNP** | **matched CG** | **p-value** |
| --- | --- | --- | --- |
|  | n=20 | n=20 |  |
| **Medicines for the treatment of** N (%)   - Polyneuropathy symptoms - Cardiovascular diseases - Metabolic diseases - Depression - Infections - Gastrointestinal diseases - Aromatase inhibitor | **50**  4 (8)  24 (48)  11 (22)  4 (8)  2 (4)  2 (8)  1 (2) | **15**  0 (0)  8 (53)  5 (33)  0 (0)  2 (13)  0 (0)  0 (0) | **0.013^1^** |
| **Chronic disabilities requiring medical treatment** N (%)   - Coronary heart disease - Arterial hypertension - Cancer in complete remission - Diabetes mellitus type II - Celiac disease - Thyroid disease - Hepatitis B - Gastrointestinal disease - Depression | **18**  0 (0)  7 (35)  1 (5)  2 (10)  1 (5)  2 (10)  2 (10)  2 (10)  1 (5) | **8**  2 (10)  2 (10)  0 (0)  0 (0)  0 (0)  2 (10)  1 (5)  1 (5)  0 (0) | **0.038^1^** |

PNP, polyneuropathy patients; matched CG, matched healthy control group; ^1^ Man-Whitney-U; significant differences are marked in bold.


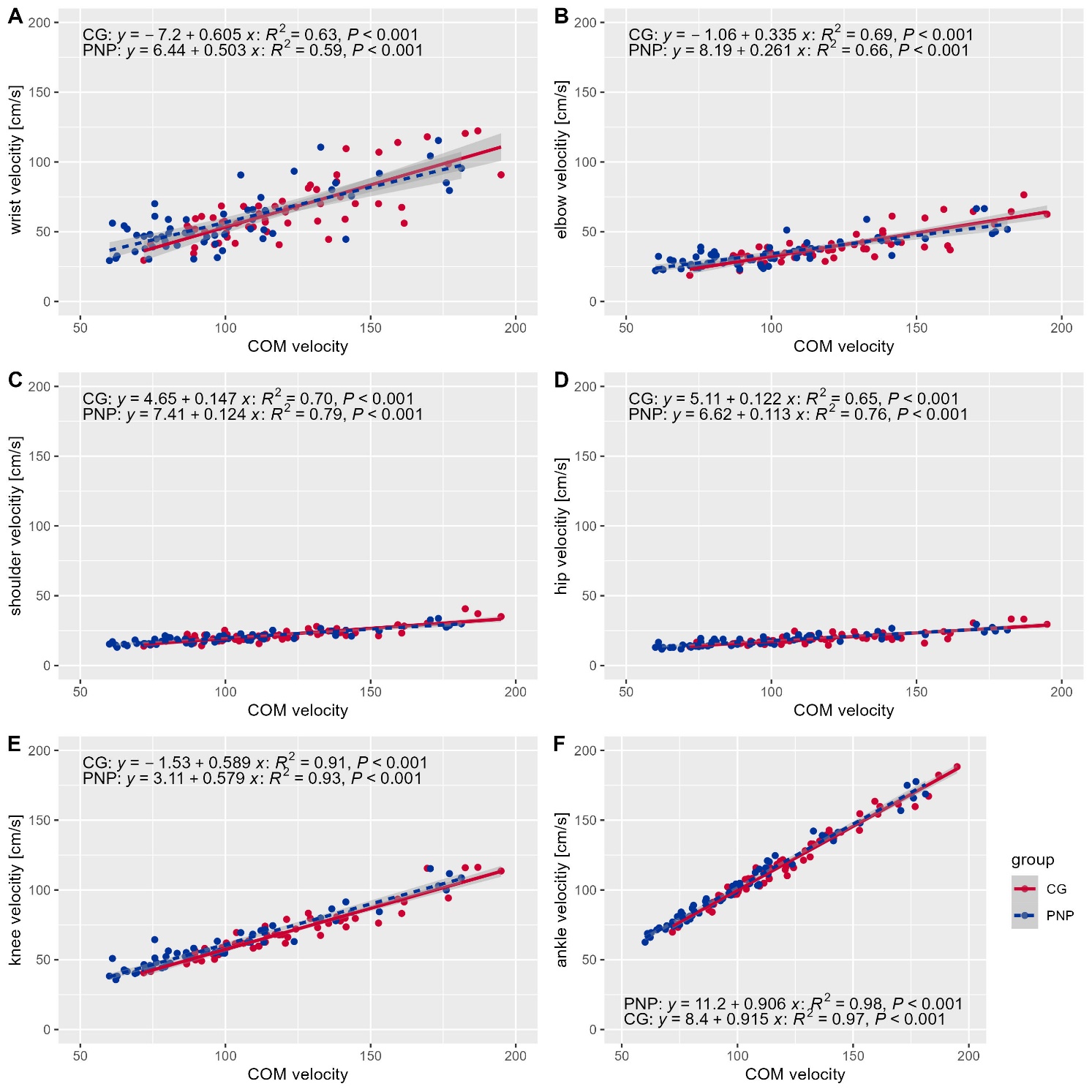


Supplement Figure 1. Correlations between center of mas (COM) representing gait speed (x-axis) and joint velocity (y-axis) across all conditions per group **(CG (red), control group; PNP (blue), patients with peripheral neuropathy), including the regression equation, R-squared and p-value, respectively.**


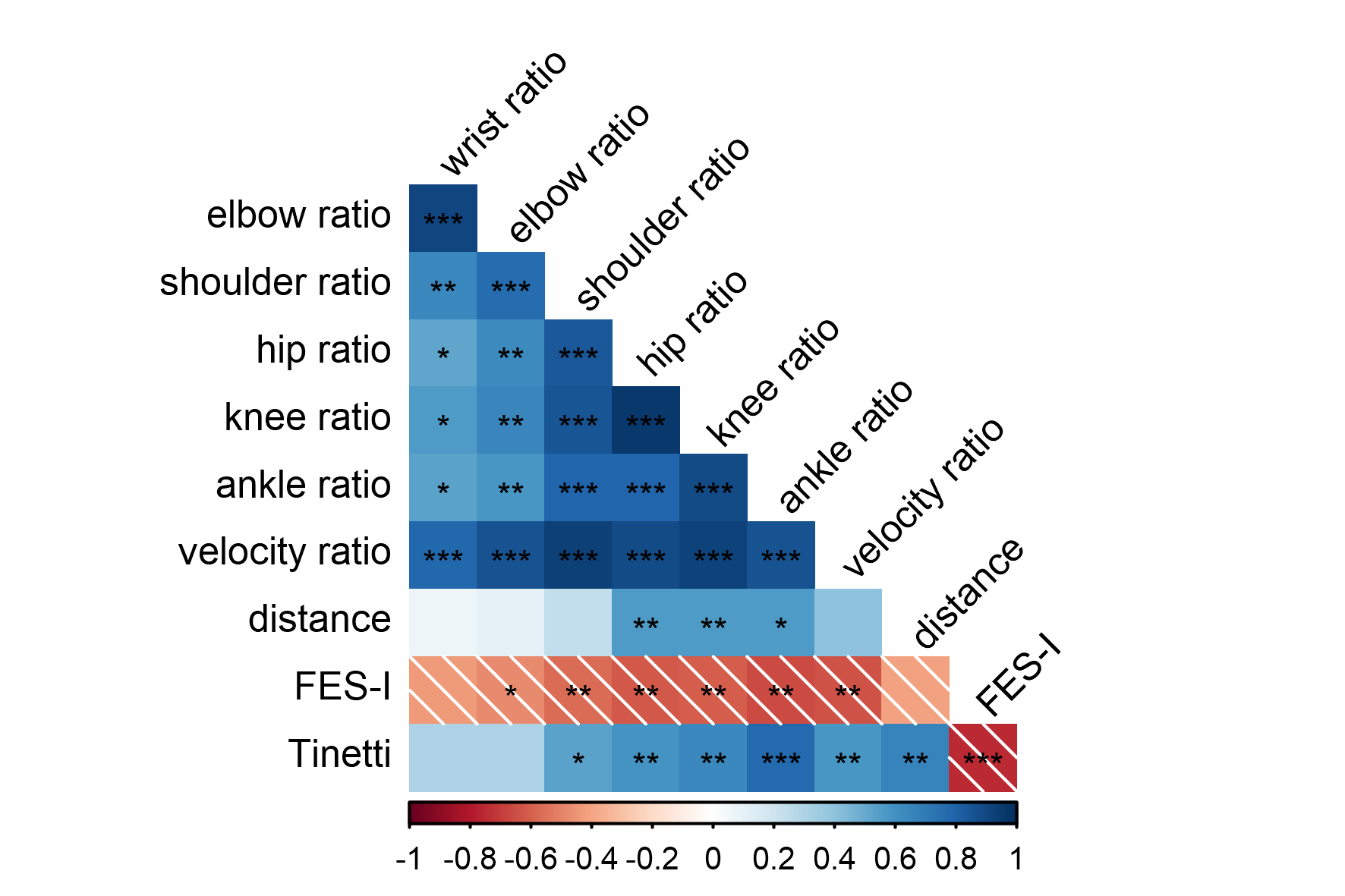


Supplement Figure 2. Correlation between joint velocity ratios (wrist-, elbow-, shoulder-, hip-, knee-, ankle ratio and overall velocity ratio) of PNP patients with maximum walking distance (distance), fear of falling (FES-I) and mobility performance (Tinetti POMA), *, p > 0.05; ** p > 0.01; ***, p > 0.001; red displays negative, blue positive correlations.
